# Design and Development Considerations of a Cyber Physical Testbed for Operational Technology Research and Education

**DOI:** 10.3390/s24123923

**Published:** 2024-06-17

**Authors:** Salaheddin Hosseinzadeh, Dionysios Voutos, Darren Barrie, Nsikak Owoh, Moses Ashawa, Alireza Shahrabi

**Affiliations:** Cyber Security and Networks, School of Computing, Engineering and Built Environment (SCEBE), Glasgow Caledonian University, Glasgow G4 0BA, UK

**Keywords:** cyber-physical systems, industrial control systems, critical infrastructures, testbed, cybersecurity education, IT/OT convergence, single-board-computer (SBC)

## Abstract

Cyber-physical systems (CPS) are vital in automating complex tasks across various sectors, yet they face significant vulnerabilities due to the rising threats of cybersecurity attacks. The recent surge in cyber-attacks on critical infrastructure (CI) and industrial control systems (ICSs), with a 150% increase in 2022 affecting over 150 industrial operations, underscores the urgent need for advanced cybersecurity strategies and education. To meet this requirement, we develop a specialised cyber-physical testbed (CPT) tailored for transportation CI, featuring a simplified yet effective automated level-crossing system. This hybrid CPT serves as a cost-effective, high-fidelity, and safe platform to facilitate cybersecurity education and research. High-fidelity networking and low-cost development are achieved by emulating the essential ICS components using single-board computers (SBC) and open-source solutions. The physical implementation of an automated level-crossing visualised the tangible consequences on real-world systems while emphasising their potential impact. The meticulous selection of sensors enhances the CPT, allowing for the demonstration of analogue transduction attacks on this physical implementation. Incorporating wireless access points into the CPT facilitates multi-user engagement and an infrared remote control streamlines the reinitialization effort and time after an attack. The SBCs overwhelm as traffic surges to 12 Mbps, demonstrating the consequences of denial-of-service attacks. Overall, the design offers a cost-effective, open-source, and modular solution that is simple to maintain, provides ample challenges for users, and supports future expansion.

## 1. Introduction

Cyber-physical systems (CPS) combine computational and physical processes to automate complex, real-world tasks, leveraging advancements in sensors, networking, and embedded computing. These systems have revolutionized various sectors by enabling more efficient, responsive, and intelligent operations. CPSs facilitate real-time monitoring and decision-making, from improving energy management in the smart grid to optimizing production processes in manufacturing. Their integration into critical infrastructures (CI) underlines their importance in ensuring operational continuity, enhancing safety, and maximising productivity and efficiency [1]. In recent years, however, there has been a notable increase in cybersecurity attacks targeting the governmental sector and CI [2]. In 2022, cyber-attacks on CPSs increased by 150% compared to the previous year [3]. The ransomware attack in May 2021 on the Colonial Pipeline highlighted the vulnerabilities of industrial control systems (ICSs), and the impact of cyber threats and emphasised seeking securing solutions [4]. Many of these attacks are due to the integration and convergence of information technology (IT) and operational technology (OT); this is known as the IT/OT convergence [5].

Over the last two decades, Supervisory Control and Data Acquisition (SCADA) systems, vital for monitoring and managing CI, have transitioned. Initially, SCADA systems were standalone and isolated, known as monolithic SCADA. With the advent of local area networks, the second generation, called Distributed SCADA, allowed systems to connect, although with proprietary limitations. The third generation, Networked SCADA, introduced universal communication protocols and TCP/IP stacks, expanding connectivity options through wide area networks. The most recent fourth generation, aligning with the principles of industry 4.0, merges traditional SCADA into industrial IoT (IIoT) and cloud technologies, greatly enhancing functionality and monitoring capabilities but also embodies the shift towards smart automation and data exchange in manufacturing technologies, characteristic of Industry 4.0 [4,6,7,8]. Traditionally, proprietary protocols could achieve security via obscurity [9]. However, the shift towards adopting well-established standards, while enhancing functionality and reducing costs, has also exposed vulnerabilities [10]. These events underscore the urgent need for enhanced cybersecurity education and research, particularly within the realm of CI, which is essential to our daily activities [11]. It is well established that performing testing on live SCADA systems is extremely dangerous as it can cause environmental damage or loss of lives. In addition, it could be costly, for instance, Nozomi Network reports shutdown event of an ICS could cost from USD 225 K up to USD 600 M [12]. Therefore, using testbeds is a more appropriate approach to address these needs, especially for the purposes of testing and validation of novel solutions [10,13]. Maynard et al. [14] list the importance of cyber-physical testbeds (CPT):

- Educational and training tools to provide practical experience in ICS security and complement theoretical knowledge with hands-on practice. It can also offer a realistic environment for training the operators;
- Research and development of new attack and defence technologies to evaluate the effect of various attack vectors and mitigation strategies;
- Enable functional testing of control systems, including network delay and other performance indicators, ensuring systems meet operational requirements;
- Facilitate vulnerability assessment, discovery, and exploitation of weaknesses in the design;
- Attack simulation and risk management through simulation of attacks and analysis of potential risks in a controlled environment;
- Development of countermeasures, forensic analysis techniques, and safety standards and promotion of best practices;
- Offer an environment for cybersecurity competitions such as capture-the-flag events and fostering skill development strategies.

Despite plethora of advantages and use cases of CPTs, they are not widely adopted, due to challenges associated with the development and maintenance of such systems [6,15]. A few of these challenges are the interdisciplinary knowledge and skills required for the development of such systems, the expensive cost of production when using proprietary hardware and software, the expensive cost of routine maintenance, long building time and finally the inherent dangers of interacting with the CPTs.

This article proposes a CPT that is developed for cybersecurity education, and research purposes with the aim of mitigating the design and development barriers. Therefore, contributing to the field of ICS cyber security:

- Proposing a simplified level-crossing (railway-crossing/grade-crossing) as the physical process.
- Design and selection of sensors and components to support a wide variety of attacks, including physical attacks.
- Multi-faceted network topology to facilitate cybersecurity education and support research.
- Providing an empirical platform for education, research, and collaboration between academia and industry.
- An open-source, modular, low-cost, and easy-to-maintain physical platform with support for future expansion.

The remainder of this article is organised as follows: Section 2 provides an analysis of existing designs and components of a CPT. Section 3 discusses the design requirements, proposes a novel CPT, and introduces the components of the proposed CPT. Section 4 pertains to the embedded vulnerabilities within the CPT. Section 5 evaluates the CPT and presents impacts of exploiting the vulnerabilities. Finally, Section 6 concludes and summarises the findings of the research.

## 2. Design Analysis of CPT Platforms

This section provides an overview of the frameworks and inspiring research and developments that have contributed to the design, development, and evaluation of this proposed CPT.

### 2.1. Design Framework

**Figure 1:**
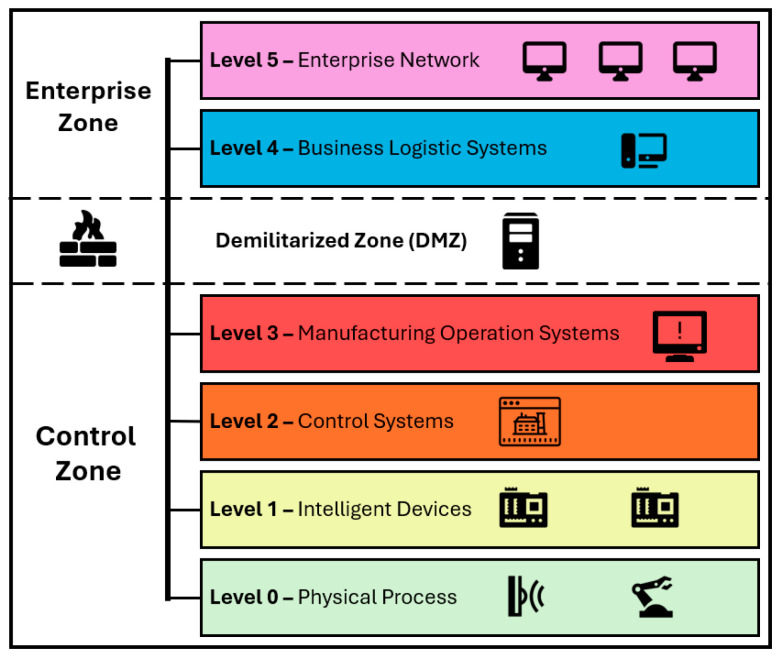
Purdue model architecture of industrial control systems.

Frank et al. [16] emphasised the importance of a systematic approach towards the design of the CPTs and have proposed a holistic but conceptual framework for the entire CPT life-cycle. In practice, however, many of the published research studies [4,6,17,18,19] that pertain to the design and development aspects of the CPTs have explicitly followed the Purdue enterprise reference architecture model (PERA) [20]. Özçelik et al. [17] have also used defence in depth for securing the CPT in addition to using PERA for structuring their proposed testbed. PERA is a framework for organizing ICSs into defined zones and levels, creating a structured hierarchy that distinguishes between operational and information technology systems. Defence in depth is a cybersecurity strategy that employs multiple layers of defence across an organization’s systems. Together, PERA’s segmentation enables the precise application of defence in depth strategies, integrating multiple security measures at each specific level and zone within a CPT to bolster resilience against cyber threats. Several researchers [4,6,15,21,22] have followed the threat modelling and cyber kill-chain for developing and testing a variety of cyber-attacks. The PERA model divides ICS into multiple zones and levels (Figure 1).

1. Enterprise zone: This zone consists of IT infrastructures such as servers and networking equipment that support enterprise-level applications and services. This zone is further divided into two levels (levels 4 and 5).
2. Demilitarized zone (DMZ): Controls the communication between the enterprise zone and the control zone of the ICS, effectively bridging between the IT and OT. DMZ is often referred to as level 3.5 [23,24].
3. Control zone: This zone is responsible for monitoring and controlling the OT systems, this includes precise control over production activities, process automation, and real-time data acquisition for operational efficiency and safety. This zone is divided into four levels:
  - Level 0: the physical process includes sensors and actuators that are sensing and making changes to the physical process as needed.
  - Level 1: intelligent devices like Programmable Logic Controllers (PLCs) that execute real-time control strategies.
  - Level 2: supervisory control systems, such as human–machine interface (HMI) and data historian.
  - Level 3: manufacturing operation systems that manage production workflow.
4. Safety zone: safety devices to monitor and manage the safety of ICS.

### 2.2. CPT Types

CPTs are generally categorized based on whether they are virtual or incorporate actual physical components.

#### 2.2.1. Virtual

These are entirely software-based and used as co-simulations for cyber and physical parts of the CPT [25]. To simulate each component of the system, they combine multiple platforms, such as DETER, Emulab, CORE, NS2/3, OPNET, OMNET++, SSFnet, and RINSE, which are used to simulate networking infrastructure [14], whereas MATLAB, Modelica, Ptolemy, PowerWorld, and PowerFactory are used for simulating the physical process [6,26]. GRFICS [15] is an example of a virtual CPT that uses a custom-made game to simulate the physical process of a chemical plant and virtualisation for the networking infrastructure.

#### 2.2.2. Physical

This type of CPT creates a scaled-down model of the real-world ICS. The largest example is the National SCADA Test Bed [27,28] with 61 miles of 138 kV power transmission lines. SwaT, EPIC, and WADI are a few other examples that have tightly integrated computational and physical components, such as sensors, actuators, and controllers, to monitor and control physical processes. These CPTs deploy real hardware and industrial-grade software [5].

#### 2.2.3. Hybrid

A hybrid CPT combines elements of virtual and physical CPTs together and takes advantage of emulation. This is the most common type as it can leverage the advantages of virtualized, emulated, and physical components using hardware-in-the-loop (HIL) [5]. Depending on the CPT application and purpose, components of the CPT such as the physical process, industrial automation, or networking might be virtualized, emulated, or implemented using HIL. LISCTER [22] is an example where the physical process is deployed, while the Programmable Logic Controller (PLC) and the SCADA systems are emulated using Raspberry Pi single-board computers (SBC).

### 2.3. Strengths and limitations of Different Types of CPTs

Each category of CPT has its advantages and disadvantages. For instance, Cintuglu et al. suggest that full virtualisation using co-simulation is the easiest way to create a CPT [29]. In addition, they are less expensive compared to physical CPTs [30]. However, the fidelity of the virtual process simulation is lower in comparison to physical deployment. As such, virtual CPTs cannot provide a visual or empirical understanding of the impacts and consequences of cyber-attacks. Finally, their performance is subjected to host’s performance, task scheduling, and availability of resources [5].

Physical CPTs are expensive and time-consuming to build, maintain, and utilise [31]. They occupy physical space and have poor mobility. They offer realism and high fidelity, but that comes at the cost of safety risk, lack of diversity, and limitations that are imposed due to safety considerations and the scale of the system [5].

Depending on the design of the hybrid CPT, it trades off between the pros and cons of the other two categories. Although, Smadi et al.’s [5] generalisation places them between the virtual and physical CPTs in terms of cost, flexibility, fidelity, and development and maintenance time.

### 2.4. ICS Components of a CPT

Regardless of the type of CPT, they all share a set of common components that are essential for the operation of the ICS. To establish the required components, a brief description of their role is provided with a full description being available in [6].

ICS field devices directly interact with the physical process; they are the sensors and actuators that provide data to controllers or receive data from the controller to execute actions. Field devices facilitate monitoring, decision-making, and action implementation.

A Programmable Logic Controller (PLC) is a microprocessor-controlled device that reads input signals, executes programmed instructions and generates output signals for industrial processes. They are equipped with a Real-Time Operations System (RTOS) for consistent execution time. They often have a web interface for easier management and support of various communication protocols.

The human–machine interface is a dedicated screen enabling operators to oversee automation processes, displaying various plant states across multiple process networks and devices. Additionally, operators can utilize the HMI to manually issue command controllers, adjusting production chain values as needed.

Data Historian is a software that is designed to gather real-time data from processes, consolidating it into a database for analysis. It ensures rapid data ingestion without loss and employs industrial interface protocols.

Supervisory Control and Data Acquisition (SCADA) systems operate at the top tier of the ICS hierarchy, overseeing and controlling centralised data from various field sites. They facilitate communication between devices and serve as remote access points for operators within the OT network.

### 2.5. Communication Protocols and Vulnerabilities

To enable communication between different components of the CPT, various communication protocols are utilised. The choice of protocols to support this communication does not seem to follow a predefined set of rules within the research community. The choice of protocols is justified by the ease of integration especially for a hybrid CPT where virtual and physical aspects are interlinked, availability of resources and their inter-compatibility, requirements of the ICS such as low-latency or wireless communication, and vulnerability to attack vectors.

Conti et al. [6] have aggregated a comprehensive list of ICS communication protocols used in CPTs, where their analysis revealed Modbus as the most common protocol with nearly a 40% share and DNP3 with nearly a 13% share being the second.

Understanding the various types of attacks in complex ICS is crucial for developing and evaluating defence mechanisms. Consequently, CPTs must be able to replicate realistic attacks. Depending on the focus and the purpose, communication protocols, architecture, and components of the CPT, a series of vulnerabilities are considered and exploits are developed. Conti et al. [6] divide the attacks into two categories of network and physical:

- The five main categories of network attacks are reconnaissance, man-in-the-middle (MitM), injection, replay, and denial of service (DoS);
- The three main categories of physical attacks are stealth, device manipulation, and direct damage.

## 3. Proposed CPT

This section provides the information and motivations behind the proposed CPT.

### 3.1. CPT Purpose and Focus

The proposed CPT is mainly intended for educational purposes and academic research in the field of cybersecurity. It targets cybersecurity practitioners who have a sound understanding of IT infrastructure, network security, and threat analysis, aiming to provide them with a comprehensive platform for honing their skills in identifying, analysing, and mitigating cyber threats in OT. It aims to provide a safe controlled environment to put their theoretical skills into practice and experience the OT environment architecture and its main components. It further supports practitioners to familiarize and identify the vulnerabilities within the ICS, exploit the vulnerabilities, observe the impact of the cyber-attacks on the physical environment, and develop, test, and validate solutions to detect and prevent the cyber-attacks.

The main targeted audience, therefore, may not have a comprehensive understanding of the differences between IT and OT systems. Thus, the CPT is designed to bridge this knowledge gap by offering immersive experiences within OT environments. By providing a simulated yet realistic OT environment, complete with its architecture and main components, the CPT allows cybersecurity practitioners to gain hands-on experience and insights into the unique challenges and vulnerabilities present in an ICS. This experiential learning approach enables practitioners to not only understand theoretical concepts but also to develop practical skills in identifying, analysing, and mitigating cyber threats specific to OT environments. Additionally, the CPT facilitates the exploration of the intricate interplay between cyber-attacks and physical consequences, empowering practitioners to devise and validate effective cybersecurity solutions tailored to safeguarding critical infrastructure and industrial systems.

### 3.2. Type of CPT

The proposed CPT uses a hybrid implementation. Hybrid CPTs have been shown to strike a balance between cost, fidelity, flexibility, and time, making them well-suited for various needs. In this design, networking fidelity takes precedence, given the CPT’s focus on cybersecurity and networking research and education, rather than control systems, automation, physical processes, and industrial safety. As a result, the CPT emphasises replicating network behaviours and security protocols accurately over physical component interaction. This prioritisation allows for thorough experimentation and analysis of cyber threats and defence mechanisms in a controlled yet realistic networking environment, tailored to the specific requirements of cybersecurity practitioners. For instance, in GRFICS multiple slave MobusTCP devices with different internet protocol (IP) addresses have identical media access control (MAC) due to them being simulated within a single virtualised environment, hence compromising on the networking realism at the cost of reduced configuration and storage requirements. Hybrid CPTs have higher networking fidelity compared to virtualized ones; additionally, certain attacks that rely on device implementation errors may not work against virtualised components [25,32].

Support for a variety of challenges is another major decision factor for the given audience. A hybrid CPT can showcase a wide range of vulnerabilities that could compromise an ICS, making it a comprehensive and effective learning tool. This includes not only common cybersecurity threats such as reconnaissance, MitM, and DoS but also more sophisticated attacks targeting specific industrial processes or components such as, coordinated covert attacks, service degradation, and system identification. A hybrid CPT with a physical deployment of its ICS process can support perception-layer, physical, and even transduction (analogue) attacks [33]. This enables practitioners to develop a deeper understanding of the evolving threat landscape and the importance of implementing robust security measures in OT environments. Furthermore, the ability to replicate real-world vulnerabilities and their potential impact on industrial operations enhances the relevance and practicality of the learning experience.

Therefore, this design takes advantage of emulation to reduce the cost of developing a physical CPT. Deploying industrial-grade equipment would increase the cost and reduce the mobility and flexibility of the CPT. Instead, industrial-grade equipment is emulated using single-board computers, which offers several advantages. Firstly, emulation allows for greater scalability, enabling the CPT to accommodate a larger number of users and experiments without the need for additional physical hardware. Secondly, it enhances the portability of the platform, facilitating deployment in different environments. Additionally, it simplifies maintenance and troubleshooting.

### 3.3. ICS Process of CPT

**Figure 2:**
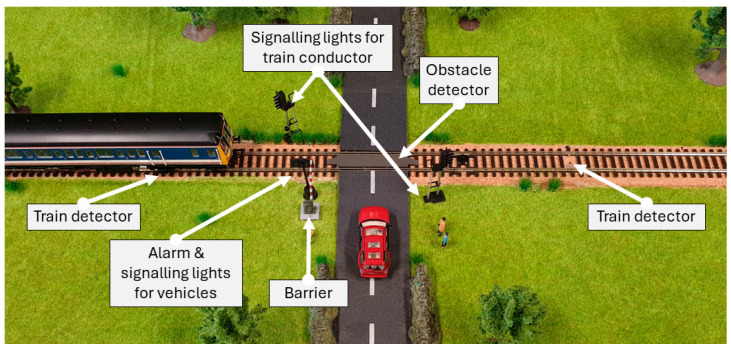
ICS process of the CPT and its sensors and actuators.

The physical implementation of the ICS process must align with the design requirements of this CPT. These requirements include ensuring safety, the ease of understanding the process, the ability to reset after each cyber-attack (recovery time), and the reproducibility of the experiments. A model of an automated level-crossing (also referred to as railroad or grade crossing) was chosen as the physical process of the CPT, this is depicted in Figure 2.

While most CPTs are related to the smart grid [26,34], they involve high-voltage components, require a considerable footprint, and can be complex to comprehend without a background in power generation and distribution systems. The safety of the practitioners during their interaction with the CPT’s process is of the utmost importance. This proposed CPT does not pose any health and safety risks to the practitioners, environment, and its components while conducting experiments, even during targeted cyber-attacks. Although the consequences of a malfunction or attack on a level-crossing concern public health and safety, in this CPT the consequences are only observational and symbolic. The choice of an automated level-crossing allows us to take advantage of model train parts, which are further modified to operate with 5 volt DC. The proposed CPT has a quick recovery time; the process can be easily reinitialised after each experiment without causing any damage to the system components. The physical deployment of the network and the physical process ensures the repeatability and consistency of the experiments. An automated level-crossing is a simple and easy-to-understand process, many have experienced and can easily relate to it, it is part of transportation system critical infrastructure, and supports a variety of physical attacks from minor delays in service to life-threatening accidents.

This simplicity ensures that practitioners can move to the second stage of the ICS cyber kill-chain and focus on understanding the ICS-specific vulnerabilities. The ICS cyber kill-chain suggests that there may be a significant lag between stage one to stage two due to prolonged tuning and testing [35] as the attackers must understand the physical process at the victim’s facility, research the built-in safety checks and redundancies specific to that victim, and develop a strategy for bypassing those obstacles to achieve their physical goal [15]. This hurdle can be removed by adopting a simple physical process such as the automated level-crossing. A significantly simplified version of the Tennessee Eastman process is virtualized in [9,15]. Green et al. [18] suggests a simple water tank system process to help practitioners understand the potentially achievable goals of an adversary, as opposed to SWaT [36] that has a complex and hard-to-maintain process. The ICS cyber kill-chain [35] has two stages, the first stage resembles the cyber kill-chain commonly seen in IT; however, its second stage is unique to ICSs, and it targets the physical systems, which involves the following:

- Develop and tune attacks to target a specific ICS for the desired impact;
- Validate attacks for their meaningful and reliable impact on a similar or identically configured system;
- Deliver the attack, where the goal is to manipulate the process to cause significant harm.

### 3.4. Architecture

**Figure 3:**
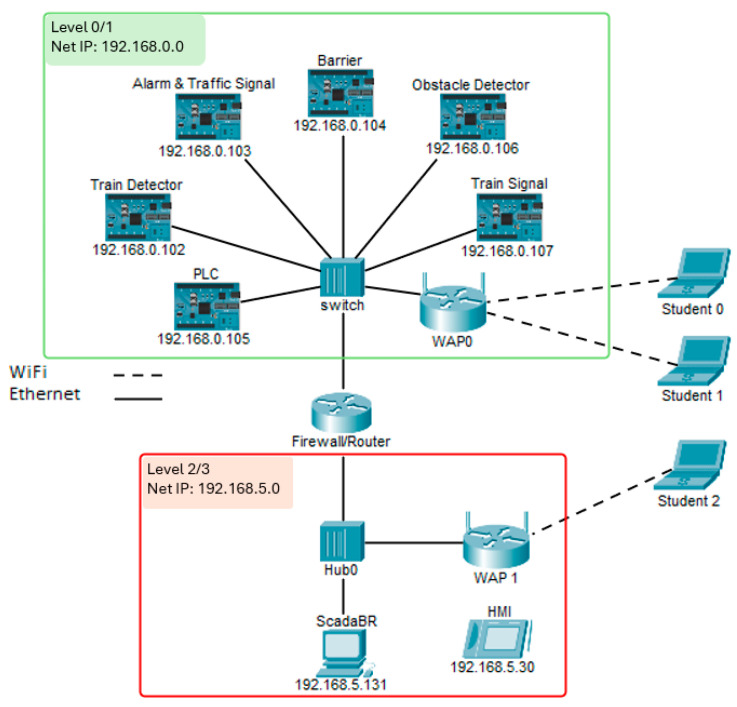
CPT architecture and network topology.

The CPT architecture follows the PERA model with a focus on the control zone that pertains to the OT. It spans level 0 to level 3 of the PERA model, with field devices in level 0, a PLC in level 1, and an HMI/Historian in Level 2/3 (Figure 3).

Inspired by the defence in depth, level 1 and level 2 are segmented using subnetting and the connection between the two layers is enabled through a firewall/router placed between the two subnets.

To facilitate the interaction of students with different layers of the CPT and exploit a full range of attacks, two wireless access points (WPA0 and WPA1) are placed within the CPT, granting direct access to each level. Learners can connect directly to one of these access points depending on their requirements and objectives.

### 3.5. CPT Components

There are certain components that are common and necessary for the operation of an ICS, and they should be replicated within a CPT. Since this CPT is aimed at skill development in OT rather than IT, the design is mainly focused on the control zone of the PERA model instead of the enterprise zone. The control zone consists of many field devices, which are the sensors and actuators.

#### 3.5.1. Actuators

Signalling lights in an automated level crossing are used to signal the vehicles about an approaching train to the level crossing and, therefore, control the flow of the vehicles. There are also signalling lights to inform the train conductor about the status of the upcoming crossing that is placed before the crossing and indicate whether the crossing is clear and safe to proceed. The same setup has been replicated on the CPT.

An automated level crossing also has barriers that physically prevent vehicles and pedestrians from crossing the tracks when a train is approaching. CPT is equipped with an automated barrier that uses a 5 volt DC motor to raise and lower the barrier arm.

Along with signalling lights and barriers, an audible alarm system is a crucial component of many automated level crossings. This alarm typically consists of bells, horns, or sirens that activate to provide an auditory warning of an approaching train. The alarm starts sounding when the lights begin to flash. An active Piezo buzzer is deployed in the CPT as the alarm.

#### 3.5.2. Sensors

The CPT has sensors to detect the train. In the real world, a train approaching the crossing is detected using circuit tracks, axel counters, radar, or light detection and ranging (LiDAR) [37]. In this CPT, photoresistors are placed under the tracks as sensors, detecting a train by the shadow it casts on the photoresistors. This method of detection is compact and inexpensive and was strategically chosen to allow the demonstration of an analogue attack on sensors (transduction attack) [38,39,40,41].

Automated level crossing employs various methods of obstacle detection on the track such as radar, LiDAR, computer vision, and inductive loop [42]. Given the scale and the cost of the CPT, a similar effect was achieved in the CPT using a magnetic hall-effect sensor, which was placed under the crossing. In conjunction with a magnetic model vehicle, they mimic the effect of an inductive loop sensor for the detection of the model vehicle on the track. Infrared receiver: an infrared receiver is incorporated into the CPT for two distinct purposes:

- Demonstrate infrared attacks in ICSs [43];
- Provide a quick mechanism to reset individual components and to reinitialize the CPT after an attack.

#### 3.5.3. Field Devices

To keep the CPT cost-efficient and flexible, industrial field devices are emulated using general purpose single board computers (SBCs) such as Raspberry Pi (RPI) and BeagleBone Black (BBB); a similar approach is adopted in [22]. In total, there are six field devices, listed in Table 1.

Modbus TCP/IP [17] was used as the communication protocol. Modbus is a widely used protocol [21] and it has several variations. The latest revision of the protocol is standardized by Modbus Organization as the Modbus/TCP Security protocol, which utilizes transport layer security (TLS) [44,45]; however, ModbusTCP/IP’s lack of security can be used for educational purposes [46]. Furthermore, it is simple to implement due to the plethora of free libraries such as pyModbusTCP [47] for Python and jmodbus [48] for Java. This CPT uses the pyModbusTCP library and Python to emulate the Modbus slave devices on the BBB.

#### 3.5.4. Programmable Logic Controller

**Figure 4:**
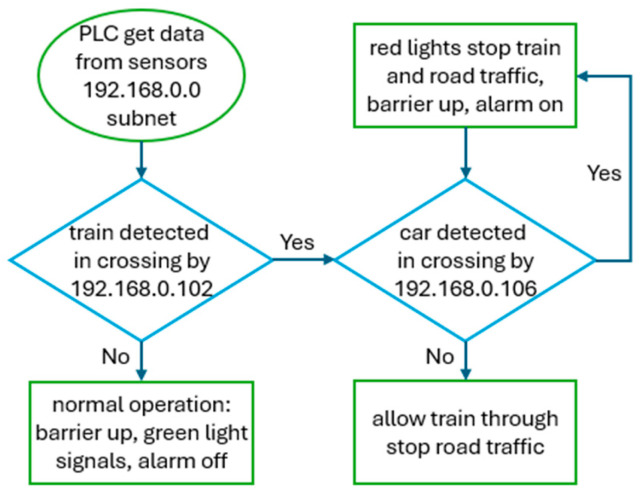
Operation flowchart of the CPT’s process and PLC’s decision making.

OpenPLC [49] is an open-source and free platform that can emulate PLCs on a variety of popular devices, and it is utilised in several other designs [9,15,22,30]. In this CPT, a Raspberry Pi 3 has been configured to act as the master device. Using the Modbus TCP, the PLC regularly sends read and write requests to gather sensor data, determine the correct state of the level crossing, and update the state of actuators to automate the level crossing, this is demonstrated in Figure 4. It also gathers all the process values and provides them to the SCADA system.

#### 3.5.5. SCADA System

**Figure 5:**
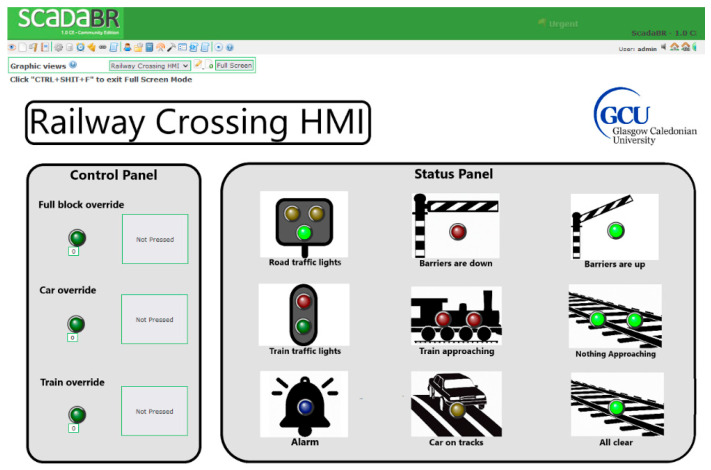
ScadaBR’s HMI of the CPT.

The ScadaBR [50] server is hosted on LattePanda x86-64 architecture. ScadaBR, which is a free and open-source platform for the development of SCADA systems, is used in this design [9,15]. Other notable solutions are PyScada, ScadaLTS, OpenSCADA, The Tango Control, and AdvancedHMI. However, ScadaBR has a variety of known vulnerabilities that can be exploited for educational purposes (further discussed in Section 4), it is accessible and operable through a web browser, and it provides a comprehensive suite of tools for storing and analysing real-time data. Modbus TCP is the communication protocol between the ScadaBR and the PLC. ScadaBR provides a web-based HMI for operators to monitor and control the processes (Figure 5). A tablet that is connected to the WAP1 serves as the interface.

#### 3.5.6. Safety Instrumentation System (SIS)

A safety instrumentation system has been implemented in the CPT. Sensors and actuators are electrically connected to an RPI Pico microcontroller. The SIS’s task is to monitor the CPT and provide safety in case of a malfunction or cyber-attack. For demonstration purposes, it can be enabled or disabled using the remote control. The SIS is air-gapped; therefore, it is not connected to the network and it is not readily configurable.

## 4. Embedded Challenges

When designing the challenges, several frameworks and resources have served as inspiration, such as the industrial cyber kill-chain, Mitre Att&ck, Open Web Application Security Project (OWASP), common vulnerability and exposure (CVE), and common weakness enumeration (CWE). This section explores the specific challenges that are developed for the CPT to achieve two primary goals: (a) offering a realistic scenario for evaluating potential security threats under controlled conditions; and (b) developing and testing strategies to improve CPS security. Overall, the platform supports a wide range of attacks, as follows.

Wireless attacks on WPA0 and WPA1 are serving as the entry points to different layers of the CPT. WPA1 is configured using a wired equivalent privacy (WEP) security protocol that uses a 40-bit shared-secret key. The secret key is staged such that it is a common word available in Kali Linux’s wordlists. This gives students the option to run a dictionary attack or crack the password to obtain the WEP key and gain quick access to the CPT through the WPA1.

Concerning network attacks, the CPT facilitates the exploration of a wide array of networking and cybersecurity principles, including but not limited to, reconnaissance techniques, replay attacks, address resolution protocol (ARP) poisoning, medium access control (MAC) spoofing, man-in-the-middle (MitM), dynamic host configuration protocol (DHCP) starvation and denial of service (DoS). This is possible thanks to the high fidelity of the CPT’s network, which is rooted in its physical implementation.

A range of OT attacks that target the ICS components are supported by the CPT, such as system identification, service degradation, coordinated–covert attacks [33] targeting the actuators and sensors with actuator enablement–disablement (AE/AD), and sensor erasure and sensor insertion (SE/SI) [51], respectively. Additionally, ICS components and protocols that have documented vulnerabilities can also be exploited, such as the CVE-2019-16344, CVE-2019-16321, and CVE-2021-26828 for the ScadaBR and CVE-2021-31630 and CVE-2018-20818 for OpenPLC. Similarly, ModBus TCP’s lack of integrity and encryption can be exploited.

Finally, Fu et al. [52] emphasise that “*Cyberphysical systems must cope with analogue threats that an adversary could exploit without any special-purpose equipment*”. This CPT provides an opportunity to demonstrate analogue transduction attacks, through targeting the infrared sensor and the photoresistors. By manipulating the lighting around the photoresistors using an external light source such as a flashlight. The infrared receiver also showcases the wireless compromise technique [53], where adversaries can gain access to and override control commands.

## 5. Evaluation of CPT

This section demonstrates four of the attack scenarios and their consequences on the proposed CPT.

### 5.1. Analogue Attacks

**Figure 6:**
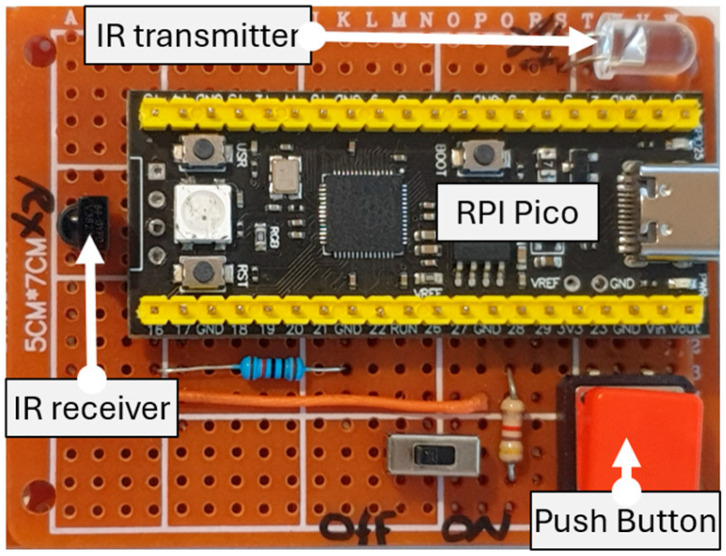
Developed RIT using RPI Pico.

To demonstrate the analogue attack on the infrared receiver, a rogue infrared transceiver (RIT) was developed using RPI Pico equipped with both an infrared receiver and transmitter, as depicted in Figure 6.

RIT is programmed to constantly scan for incoming infrared signals. In this scenario, it is assumed that automated level-crossing barriers are overridden for inspection and maintenance purposes using a legitimate remote control. The RIT successfully captured and stored the infrared signals. At this point, RIT is armed, and pressing the push button initiates a replay attack that lifts the barriers. In another scenario, an analogue attack targeted the train detection sensors. By shining light onto these sensors using a flashlight, the train detection mechanism was compromised. This failure led to the PLC’s inability to activate the safety routines of the automated level-crossing, thereby impacting the CPT’s operational safety. The issue stemmed from the deliberate use of photoresistors in the detection mechanism, which depends on detecting the train’s shadow as it passes over the sensors. Consequently, the PLC did not receive accurate information to implement the essential process control routines needed to secure the crossing.

### 5.2. DoS Attack

**Figure 7:**
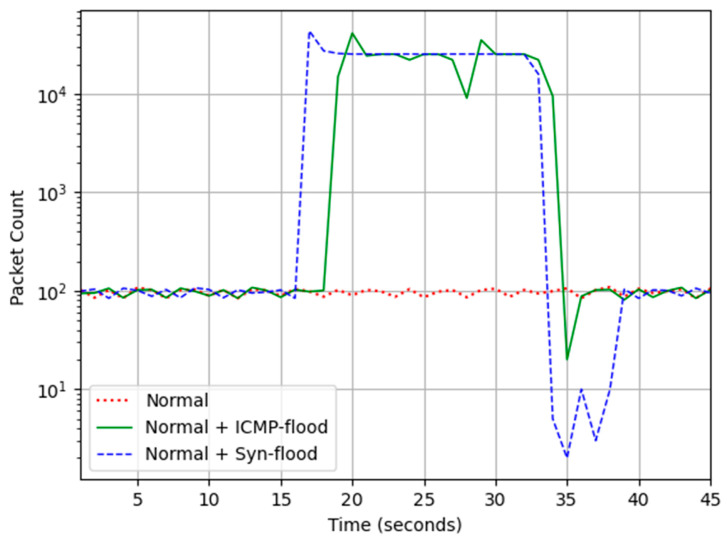
Packet rate during the attacks.

During the regular operation of the CPT, approximately 100 packets are exchanged every second; this is demonstrated in Figure 7.

**Figure 8:**
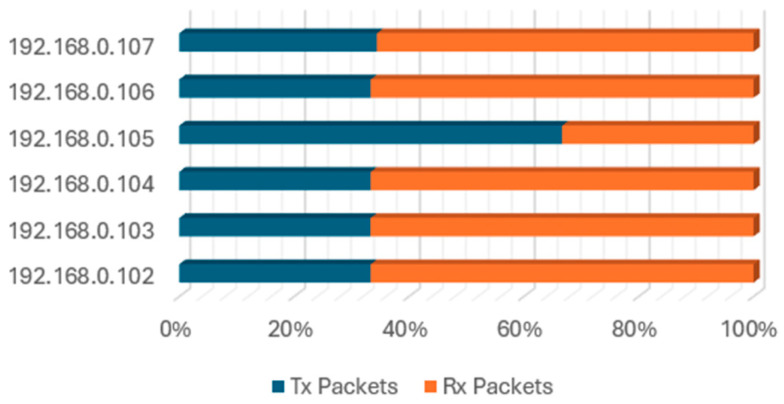
Packet exchange ratio of control zone components.

The bulk of the traffic pertains to the communication between the client (master) PLC (192.168.0.105) and the rest of the Modbus slaves, as depicted in Figure 8. The CPT has stagnant network traffic, with the PLC regularly issuing the Modbus requests and the servers (slave devices) providing the response. The PLC pools all the servers within 0.2 s.

**Figure 9:**
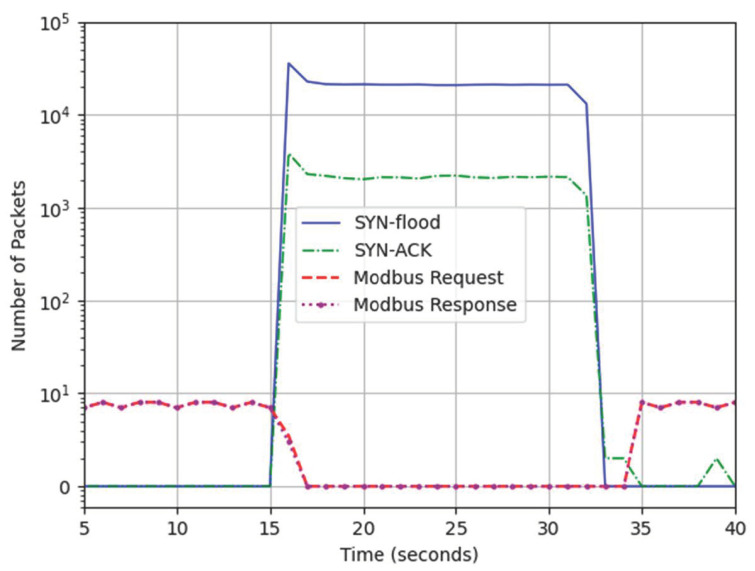
Impact of Syn-flood attack on Modbus TCP.

Two denial-of-service (DoS) attack scenarios were considered. First, the Sync flood attack targeted the train detector (192.168.0.102) Modbus TCP port 502. The second scenario was the ICMP flood attack that targeted the PLC. The number of Sync packets per second surged to 21,000, as depicted in Figure 9. This overwhelmed the Modbus server as it was able to respond with a Syn-Ack to only 10% of the Syn packets. The Modbus connection was completely lost within the first second of the attack and it was recovered 2 to 3 s after the attack was stopped. This resulted in the PLC not noticing the arrival of the train and failing to activate the process control routines.

In the second scenario, the ICMP echo request (ping) packets flooded the PLC; this influx overwhelmed the device, rendering it unable to communicate with the rest of the devices for the duration of the attack. Hence, the PLC did not deploy the necessary process control routines to secure the crossing. Both scenarios led to a collision between the train and the vehicle. Once the ICMP attack was stopped, the CPT was able to recover within a second and resume normal operations. Both attack scenarios generated a traffic of 12 Mbps, which was enough to overwhelm the Modbus client and server; however, it was noted that the Modbus communication was able to recover faster from the ICMP compared to Syn. This emulated devices are exhibiting the vulnerabilities of legacy devices that possess limited resources and are prone to overloading, thus underscoring the preference for passive versus active network scans [54].

### 5.3. Attack on SCADA System

This scenario involved an arbitrary code execution vulnerability in ScadaBR, documented as CVE-2021-26828, resulting from the unrestricted upload of dangerous-type files (CWE-434). ScadaBR uses JavaServer Pages (JSP) to generate dynamic web applications on a web server’s backend. Users were expected to upload an image on this targeted system. However, due to unrestricted file upload and lack of validation, a JSP reverse-shell payload was uploaded instead, establishing a connection to the adversary’s device. This breach enabled access to a command line interface with elevated privileges on ScadaBR host devices, facilitating the execution of shell commands or code on the target machine. Consequently, this allowed for the exfiltration of information from the compromised device.

### 5.4. Coordinated Attack

These attacks focus on the second stage of the ICS cyber kill-chain, necessitating comprehensive knowledge of the entire system [33], and can manifest in various forms such as service degradation and covert attacks. One such scenario involved simultaneously targeting two field devices and the HMI with AE/AD and MitM attacks, respectively. While a vehicle was stuck on the crossing, the train signalling lights and alarm were disabled using the AD attack, allowing the train to continue.

However, the attack did not end there; an operator could notice the obstacle detection warnings on the HMI as unusual behaviour. To prevent this, a MitM attack was executed concurrently, obscuring the warnings from the PLC to ScadaBR and its HMI, resulting in a covert physical attack that led to the train colliding with the obstructing vehicle.

**Figure 10:**
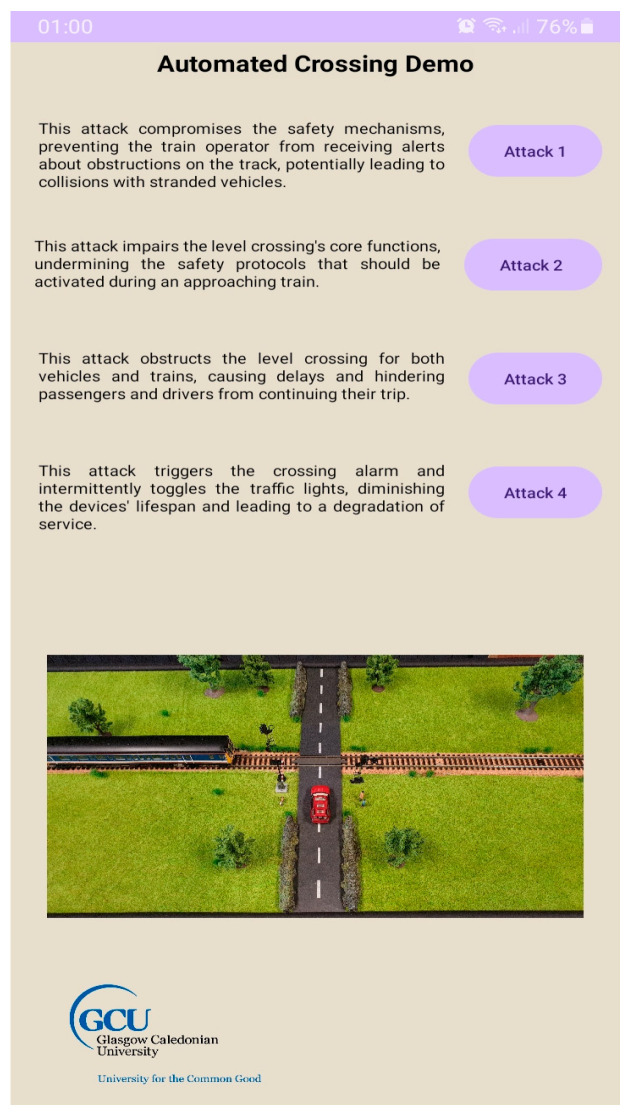
Android application to demonstrate attacks on actuators.

Finally, to readily demonstrate attacks on the Modbus protocol and their consequences on the CPT, jmodbus was used in the development of an android application, Figure 10. This application targets actuators within the CPT from a smartphone and executes an AE/AD attack to undermine the safe operation of the process.

## 6. Conclusions and Future Work

To facilitate research and training in the realm of OT, a hybrid CPT was proposed based on a model of automated level-crossing. A balance between the cost and fidelity of the CPT was achieved by emulating expensive ICS components with general-purpose SBCs. One of the advantages of this CPT is the utilization of open-source solutions for the components, resulting in a modular design that makes future upgrades and switching to HIL feasible. Selecting specific sensors has also highlighted the CPT’s susceptibility to analogue attacks without requiring specialized equipment. Additionally, flooding attacks on the SBCs have led to complete DoS.

Choosing an automated level-crossing as the main process control system ensured safe interaction with the CPT while its simplicity allowed the practitioners to focus on the second stage of ICS cyber kill-chain and analyse potential objectives that adversaries could achieve through coordinated attacks. Consequently, the proposed CPT offers an effective, scalable, and adaptable platform that meets the design objectives.

This CPT was currently used for research and development purposes to create an intrusion detection and prevention system (IDPS), leveraging advancements in artificial intelligence and machine learning. The methodology involves simulating attacks, collecting network traffic, and crafting a specialised and tailored bespoke IDPS solution to safeguard the industrial zone of this CPT.

We are establishing collaboration with industrial partners to supplement their OT training program. There are future industrial collaboration plans to bolster the CPT’s defensive mechanisms against cyber-attacks, using cutting-edge technologies and equipment. Finally, the CPT will undergo rigorous testing and evaluation for education purposes with students who are specializing in cybersecurity and networking. This evaluation will assess the CPT’s efficacy as an educational tool, highlighting its strengths and pinpointing any weaknesses that may need further improvement in future versions.

## Figures and Tables

**Table 1:** List of field devices and their description.

Field Device	Description
Alarm and signalling lights for vehicles	LEDs and active buzzer controlled by Python code running on BBB. Visual and audible signal for vehicles/pedestrians of an incoming train
Signalling lights for train conductor	LEDs controlled by Python code running on BBB. Signalling the train conductor about the status of the crossing
Barrier	5 volt DC motor controlled by Python code running on BBB. Raising/lowering the barriers to stop the vehicle
Train detector	Photoresistors sensing the arrival of the train through a Python code running on BBB
Obstacle detector	Reed switch sensing obstacles on the crossing through a Python code running on BBB
Remote control	Infrared receiver controlled by MicroPython code running on RPI Pico. Receiving commands from a remote control for raising/lowering the barrier manually

## Data Availability

Data are contained within the article.

## References

[1] Serpanos D. (2018). The Cyber-Physical Systems Revolution. Computer.

[2] Saeed S., Altamimi S.A., Alkayyal N.A., Alshehri E., Alabbad D.A. (2023). Digital Transformation and Cybersecurity Challenges for Businesses Resilience: Issues and Recommendations. Sensors.

[3] Ginter A., Hale G., Machtemes R., Molina D.J., Wallhof M., Schneider C. (2023). 2023 Threat Report—OT Cyberattacks With Physical Consequences. https://waterfall-security.com/ot-insights-center/ot-cybersecurity-insights-center/2023-threat-report-ot-cyberattacks-with-physical-consequences/.

[4] Ekisa C., Briain D.Ó., Kavanagh Y. An open-source testbed to visualise ics cybersecurity weaknesses and remediation strategies–a research agenda proposal. Proceedings of the 2021 32nd Irish Signals and Systems Conference (ISSC).

[5] Smadi A.A., Ajao B.T., Johnson B.K., Lei H., Chakhchoukh Y., Al-Haija Q.A. (2021). A Comprehensive survey on cyber-physical smart grid testbed architectures: Requirements and challenges. Electronics.

[6] Conti M., Donadel D., Turrin F. (2021). A survey on industrial control system testbeds and datasets for security research. IEEE Commun. Surv. Tutor..

[7] Babayigit B., Abubaker M. (2023). Industrial internet of things: A review of improvements over traditional scada systems for industrial automation. IEEE Syst. J..

[8] Folgado F.J., Calderón D., González I., Calderón A.J. (2024). Review of Industry 4.0 from the Perspective of Automation and Supervision Systems: Definitions, Architectures and Recent Trends. Electronics.

[9] Ekisa C., Briain D.Ó., Kavanagh Y. VICSORT-A Virtualised ICS Open-source Research Testbed. Proceedings of the 2022 Cyber Research Conference-Ireland (Cyber-RCI).

[10] Christiansson H., Luiijf E. Creating a European SCADA security testbed. Proceedings of the International Conference on Critical Infrastructure Protection.

[11] Nankya M., Chataut R., Akl R. (2023). Securing Industrial Control Systems: Components, Cyber Threats, and Machine Learning-Driven Defense Strategies. Sensors.

[12] Nozomi Networks (2022). The Cost of OT Cyber Security Incidents. https://www.nozominetworks.com/blog/the-cost-of-ot-cyber-security-incidents.

[13] Hahn A., Kregel B., Govindarasu M., Fitzpatrick J., Adnan R., Sridhar S., Higdon M. Development of the PowerCyber SCADA security testbed. Proceedings of the Sixth Annual Workshop on Cyber Security and Information Intelligence Research.

[14] Maynard P., McLaughlin K., Sezer S. An open framework for deploying experimental scada testbed networks. Proceedings of the 5th International Symposium for ICS & SCADA Cyber Security Research 2018.

[15] Formby D., Rad M., Beyah R. Lowering the barriers to industrial control system security with GRFICS. Proceedings of the 2018 USENIX Workshop on Advances in Security Education (ASE 18).

[16] Frank M., Leitner M., Pahi T. Design considerations for cyber security testbeds: A case study on a cyber security testbed for education. Proceedings of the 2017 IEEE 15th Intl Conf on Dependable, Autonomic and Secure Computing, 15th Intl Conf on Pervasive Intelligence and Computing, 3rd Intl Conf on Big Data Intelligence and Computing and Cyber Science and Technology Congress (DASC/PiCom/DataCom/CyberSciTech).

[17] Özçelik İ., Iskefiyeli M., Balta M., Akpinar K.O., Toker F.S. Center energy: A secure testbed infrastructure proposal for electricity power grid. Proceedings of the 2021 International Conference on Information Security and Cryptology (ISCTURKEY).

[18] Green B., Lee A., Antrobus R., Roedig U., Hutchison D., Rashid A. Pains, gains and PLCs: Ten lessons from building an industrial control systems testbed for security research. Proceedings of the 10th USENIX workshop on cyber security experimentation and test (CSET 17).

[19] Gao H., Peng Y., Jia K., Dai Z., Wang T. The design of ics testbed based on emulation, physical, and simulation (eps-ics testbed). Proceedings of the 2013 Ninth International Conference on Intelligent Information Hiding and Multimedia Signal Processing.

[20] Williams T.J. (1994). The Purdue enterprise reference architecture. Comput. Ind..

[21] Chromik J.J., Remke A., Haverkort B.R. (2018). An integrated testbed for locally monitoring SCADA systems in smart grids. Energy Inform..

[22] Sauer F., Niedermaier M., Kießling S., Merli D. (1910). LICSTER—A low-Cost ICS Security Testbed for Education and Research: A Preprint. https://arxiv.org/pdf/1910.00303.pdf.

[23] Wiboonrat M. Cybersecurity of Industrial Automation and Control System (IACS) Networks in Biomass Power Plants. Proceedings of the 2023 IEEE 32nd International Symposium on Industrial Electronics (ISIE).

[24] Garton D. (2022). Purdue Model Framework for Industrial Control Systems & Cybersecurity Segmentation. US Dep. Energy.

[25] Reaves B., Morris T. (2012). An open virtual testbed for industrial control system security research. Int. J. Inf. Secur..

[26] Geng Y., Wang Y., Liu W., Wei Q., Liu K., Wu H. (2019). A survey of industrial control system testbeds. IOP Conference series: Materials Science and Engineering.

[27] U.S. Department of Energy (2023). National SCADA Test Bed. https://www.energy.gov/oe/national-scada-test-bed.

[28] Barnes K., Johnson B. (2009). National SCADA Test Bed Substation Automation Evaluation Report. https://inldigitallibrary.inl.gov/sites/sti/sti/4374057.pdf.

[29] Cintuglu M.H., Mohammed O.A., Akkaya K., Uluagac A.S. (2016). A survey on smart grid cyber-physical system testbeds. IEEE Commun. Surv. Tutor..

[30] Alves T., Das R., Werth A., Morris T. (2018). Virtualization of SCADA testbeds for cybersecurity research: A modular approach. Comput. Secur..

[31] Almalawi A., Tari Z., Khalil I., Fahad A. SCADAVT-A framework for SCADA security testbed based on virtualization technology. Proceedings of the 38th Annual IEEE Conference on Local Computer Networks.

[32] Siaterlis C., Genge B., Hohenadel M. (2013). EPIC: A testbed for scientifically rigorous cyber-physical security experimentation. IEEE Trans. Emerg. Top. Comput..

[33] de Sá A.O., da Costa Carmo L.F.R., Machado R.C.S. (2017). Covert attacks in cyber-physical control systems. IEEE Trans. Ind. Inform..

[34] Holm H., Karresand M., Vidström A., Westring E. A survey of industrial control system testbeds. Proceedings of the Secure IT Systems: 20th Nordic Conference, NordSec 2015.

[35] Assante M.J., Lee R.M. (2015). The industrial control system cyber kill chain. SANS Inst. InfoSec Read. Room.

[36] Mathur A.P., Tippenhauer N.O. SWaT: A water treatment testbed for research and training on ICS security. Proceedings of the 2016 International Workshop on Cyber-Physical Systems for Smart Water Networks (CySWater).

[37] Zawodny M., Kruszyna M., Szczepanek W.K., Korzeń M. (2023). A new form of train detection as a solution to improve level crossing closing time. Sensors.

[38] Gao M., Zhang L., Shen L., Zou X., Han J., Lin F., Ren K. (2023). Exploring Practical Acoustic Transduction Attacks on Inertial Sensors in MDOF Systems. IEEE Trans. Mob. Comput..

[39] Gao M., Zhang L., Shen L., Zou X., Han J., Lin F., Ren K. KITE: Exploring the practical threat from acoustic transduction attacks on inertial sensors. Proceedings of the 20th ACM Conference on Embedded Networked Sensor Systems.

[40] Yan C., Shin H., Bolton C., Xu W., Kim Y., Fu K. Sok: A minimalist approach to formalizing analog sensor security. Proceedings of the 2020 IEEE Symposium on Security and Privacy (SP).

[41] Tu Y., Tida V.S., Pan Z., Hei X. Transduction Shield: A Low-Complexity Method to Detect and Correct the Effects of EMI Injection Attacks on Sensors. Proceedings of the 2021 ACM Asia Conference on Computer and Communications Security.

[42] Railway B. (2019). Comparative Study of Technologies for the Detection of Obstacles in Level Crossings. http://begiralerailway.com/wp-content/uploads/2019/06/Comparative-study-of-technologies-for-the-detection-of-obstacles-in-level-crossings-v2.pdf.

[43] Schneier B. (2008). Hacking Polish Trams. https://www.schneier.com/blog/archives/2008/01/hacking_the_pol.html.

[44] Modbus Organization (2018). Modbus/TCP Security Protocol Specification.

[45] Martins T., Oliveira S.V.G. (2022). Enhanced Modbus/TCP security protocol: Authentication and authorization functions supported. Sensors.

[46] BChen, Pattanaik N., Goulart A., Butler-Purry K.L., Kundur D. Implementing attacks for modbus/TCP protocol in a real-time cyber physical system test bed. Proceedings of the 2015 IEEE International Workshop Technical Committee on Communications Quality and Reliability (CQR).

[47] (2024). pyModbusTCP Contributors, pyModbusTCP. https://pypi.org/project/pyModbusTCP/.

[48] Tzook T. (2024). Jmodbus. https://plugins.gradle.org/plugin/io.github.tomtzook.gradle-cmake.

[49] Alves T.R., Buratto M., De Souza F.M., Rodrigues T.V. OpenPLC: An open source alternative to automation. Proceedings of the IEEE Global Humanitarian Technology Conference (GHTC 2014).

[50] ScadaBR. http://www.scadasoftware.net/software/scadabr.html.

[51] Carvalho L.K., Wu Y.-C., Kwong R., Lafortune S. (2018). Detection and mitigation of classes of attacks in supervisory control systems. Automatica.

[52] Fu K., Xu W. (2018). Risks of trusting the physics of sensors. Commun. ACM.

[53] The MITRE Corporation (2023). Wireless Compromise—Technique T0860—ICS|MITRE ATT&CK®.

[54] Duggan D., Berg M., Dillinger J., Stamp J. (2005). Penetration Testing of Industrial Control Systems.

